# Development of a computer-aided design software for the quantitative evaluation of aesthetic damage

**DOI:** 10.1371/journal.pone.0226322

**Published:** 2019-12-18

**Authors:** Nelson Massanobu Sakaguti, Mário Marques Fernandes, Luiz Eugênio Nigro Mazzilli, Juan Antonio Cobo Plana, Fernanda Capurucho Horta Bouchardet, Rogério Nogueira de Oliveira

**Affiliations:** 1 Forensic Dentistry, Department of Social Dentistry, University of Sao Paulo, Sao Paulo, Brazil; 2 Institute of Legal Medicine, Zaragoza, Aragón, Spain; Universidade Estadual de Ciencias da Saude de Alagoas, BRAZIL

## Abstract

Concerns associated with the assessment of aesthetic damage or injury raise critical difficulties, such as the scarcity of methodology and standardization that may result in fundamental precepts to establish impartial forms of compensation and aiming the total reparation of bodily injury. The complexity of the aesthetic damage evaluation is associated with the confluence of legal and technical perspectives and expert subjectivity while conducting examination and writing a report. Experts face additional difficulties associated with the objectivity while assessing aesthetic damage, independently on its location or expert skills, due to complex details observed in these lesions. Another situation in the clinical area, doctors (mainly plastic surgeons) and dentists could show the improvement or not, of the aesthetic condition to the patients. In health related areas, the use of information technology has contributed to increase the number of appropriate diagnoses, besides promoting quality, efficiency and satisfaction to health care providers. In order to make this assessment more objective, a technological tool was developed to aid experts in the evaluation of aesthetic damage and report elaboration. The objective was to develop computer-aided design software for aesthetic damage quantification/evaluation that is accessible via internet to be applied as a complementary report on body aesthetic damage. The software uses as a parameter the AIPE method, translated transculturally from Spanish to Portuguese and English. The present study allowed the construction of open access auxiliary software for the evaluation of corporal aesthetic damage. Its use is facilitated by intuitive and interactive filling, and the text may be customized by the user. It transforms the report into PDF and saves all evaluations already done in its own file. Information is encrypted for added security and confidentiality. The software is available on website at https://www.aestheticdamage.com.

## Introduction

In order to establish adequate forms of financial compensation for integral reparation of bodily injury, it is fundamental to standardize concepts, methods and language [1]. The assessment of the aesthetic damage specifically, which represents a portion of corporal damages, is presented by a forensic expert who examines the injured person and writes a report with his/her considerations. Based on this report, a judge will arbitrate and determine the financial compensation [2].

The complexity of aesthetic damage evaluation is related to the confluence of medical and dental forensic perspectives, resulting in the issuance of the forensic expert report. The valuation of aesthetic damage must be defined by criteria that clearly estimate the effect that this alteration of external appearance causes on the injured person and how other individuals perceive it. The same criteria must present a terminology that is comprehensible to the authorities of law. A thorough examination and proper description and recording of aesthetic changes must be carried out. In addition, photographs must be taken with a forensic scale [3]. Standardization is essential, since one must be submitted to the same pattern of aesthetic damage evaluation regardless of the forensic expert competence or the nature of the exam site.

"Aesthetic damage" means any change to aesthetic property, harmony or bodily symmetry. Aesthetic property concept is attributed to every characteristic that an individual had before suffering the damage referring to beauty, harmony, capacity for relation and attraction, self-esteem, etc. Aesthetic damage may be categorized as temporary or permanent. In order to assess adequately the aesthetic damage, it is fundamental to consider the characteristics of the affected person and the constituent elements of the damage such as location, shape, morphology, dimensions, orientation and coloration [4].

The forensic expert must assess the severity of the aesthetic damage / injury considering that he/she has to differentiate an altered physiological substrate from ugliness of the image. Beauty and ugliness are eminently subjective standards, although it is undeniable that there are sociocultural factors that define, in different times and places, what is beautiful and what is ugly. Therefore, the forensic expert or technical assistant may establish parameters of assessment (affected area, location, perceptibility, exteriorization, and how the victim experiences the aesthetic damage), as well as take into account the personal circumstances of the victim in order to determine the magnitude of the damage. It is worth noting that each country has its own rules on how the aesthetic damage settlement is regulated, and also if there is any dismemberment between physiological and aesthetic damage, in order to avoid double quantification [5].

Cobo Plana (2010) elaborated a method to evaluate the aesthetic impairment denominated AIPE (in Spanish). In English language, it is translated as “aesthetic impairment impression analysis” (AIIA). The application of this method (AIPE) in the Brazilian context has already been reviewed and discussed [6]. This study determined that in the cases of aesthetic damage evaluation in the civil law sphere or on the degree of deformity in the criminal procedure, AIPE method facilitates the adoption of a criterion of intensity or severity on this aesthetic impairment and on the possible deformity. Moreover, this approach is easily followed by forensic experts, doctors, dentists or legal professionals [6].

AIPE method consists of four tables; the first one contains the five key questions that should be answered successively. For aesthetic damage evaluation, this approach employs five increasing levels of perception for the observer, beginning with the proof of damage and finishing with the type of emotion it causes. In this case, as each level advances, the degree of aesthetic damage increases on a scale of growing severity [5]. The method proposes the following questions:

- Is it possible to perceive the alteration of the person's image?
- Does our sight or other senses tend to focus specifically on this alteration of the person's image?
- When we recollect our patient, do we describe him/her based on the person's image change?
- Does the aesthetic damage cause any emotion to the injured person, such as sadness or similar emotions?
- If one was a family member or a person close to the injured person, could the image change affect their relationship?

In 2016, Fernandes et al. published the validation of the AIPE method. This approach suffered a cross-cultural translation form Spanish to Brazilian Portuguese. The authors applied the method to dental clinicians and postgraduate students in Legal Dentistry, thus verifying its efficiency by employing objective parameters and its reliability in assessing aesthetic damage [6].

The difficulty of impartially assessing aesthetics may be supported by the use of software applications [7]. The perception of applicability, convenience and ease of use by users are determining factors that encourage users to accept information technologies [8].

Blumenthal et al. (2011) evaluated researches employing information technology in health related sciences and confirmed that clinical decisions based on technology support decreased the amount of time spent on analyses and also the cost, resulting in a positive impact in both efficiency and quality of services [9]. According to Rogers et al. and Sander et al., internet-delivered and internet-based health works consist on a good way to reach distant communities that do not have access to certain types of health services [10–11].

Finally, a study conducted by Dahlback in 2018 concluded that there is no ideal method to evaluate the aesthetic result after surgery, and emphasizes the importance to presenting the results with regard to the patient's health and quality of life [12].

## Objective

To develop an open access software available through the internet as an auxiliary means to generate technical reports of aesthetic damage in an interactive way, based on predefined input data and user inputs.

## Materials and methods

### Tools, resources and technical aspects

The technical development of the software was assigned to the School of Engineering of São Carlos—EESC at the University of São Paulo—USP.

For the project completion, public domain resources common to open access software development medium were employed.

In view of the proposal to allow access to the system to anyone with an interest in the tool, it is available as open source software on the Internet through the domain https://www.aestheticdamage.com [13]. Website IP: 107.180.4.132.

#### Software development life cycle

The web application development project was conducted under the long-established software development methodology called "Waterfall", thus being structured in 5 phases:

1. **System requirements analysis**
2. **Design of the Application (Design)**
3. **Development (Implementation)**
4. **Testing**
5. **Operation (Post Implementation / Go Live)**

These phases were organized according to the following schedule. At Table 1, the predicted execution of each phase is illustrated by the blue-filled cells and the actual execution is demonstrated by the character 'X'.

**Table 1:** Execution chronogram in weeks x phases.

PHASE/WEEK	W1	W2	W3	W4	W5	W6	W7	W8	W9	W10	W11	W12	W13	W14	W15	W16	W17	W18	W19	W20	W 21
Requirements	**X**	**X**																			
Design			**X**	**X**																	
Implementation					**X**	**X**	**X**	**X**	**X**	**X**	**X**	**X**									
Testing													**X**	**X**	**X**	**X**	**X**	**X**	**X**	**X**	
Go live																					**X**

A delay that occurred at the development phase of the software due to tests and adjustments caused impact in the following phases (post implementation / operation).

The phase called Go Live represents the effort to put the system in a productive environment (Web) as open access platform. This phase occurred after the successful completion of the implementation, in order to ensure the availability and continuous maintenance of the application.

The documentation produced by each one of these phases is presented below.

### 1. System requirements

#### 1.1 Functional requirements

a) Creation of a new report

Implement functionality concerning the creation of a report that follows the AIPE method. The user must answer the questions and after completion, the system will issue a score regarding the aesthetic damage.

b) Report / reports history management

Implement functionality so that the user may access and manage previously performed evaluations. The reports history management screen should be organized by the patient's name, date of the examination and date / time of final report issuance. The user should be able to exclude previous reports with his/her confirmation.

c) Customization of the report content

After performing the evaluation, the system should enable the user to customize the final text of the report.

d) Exportation of the report in PDF format

Implement functionality to generate PDF file based on the report final version. This feature should be available at the report management screen.

e) Functional requirements not available

It is not part of the functional requirements of this application: graphic customization of the report, such as header or watermark addition.

#### 1.2 Non-functional requirements

a) Password cryptography

The passwords of registered users in the application should be stored with irreversible encryption, so that the authentication system validates the user's identity through the checksum of this password.

b) SSL safe communication

All communication with the application on the server must occur in encrypted form through the Hyper Text Transfer Protocol (HTTP) with SSL (Secure Socket Layer), also known as HTTPS.

c) Non-functional requirements no available

Not part of the non-functional requirements:

1. Cache storage interface

The system is not available for offline use. It does not store the information for later use while completing evaluations. In case of an evaluation that was not completely filled, the information entered will be lost and the form must be filled again.

2. Use of cookies

The system must rely solely on the password authentication mechanism to identify the user. All user-level settings are hosted on the server application.

### 2. Design of the application

#### 2.1 Use cases

The Uniform Modeling Language (UML) defines some patterns of diagrams to systematize software development. One of these diagrams, called “Use Case Diagram”, derives from the specification of the requirements and describes in an objective way the available functionalities that the user may employ to interact with the system.

Note: UML may establish other diagrams, such as Class Diagram, Sequence, Communication and Machine Status. All of them are part of the Software Development Methodology and are intended to create a unique language so that any developer or analyst can communicate and understand the operation of this system.

**Fig 1:**
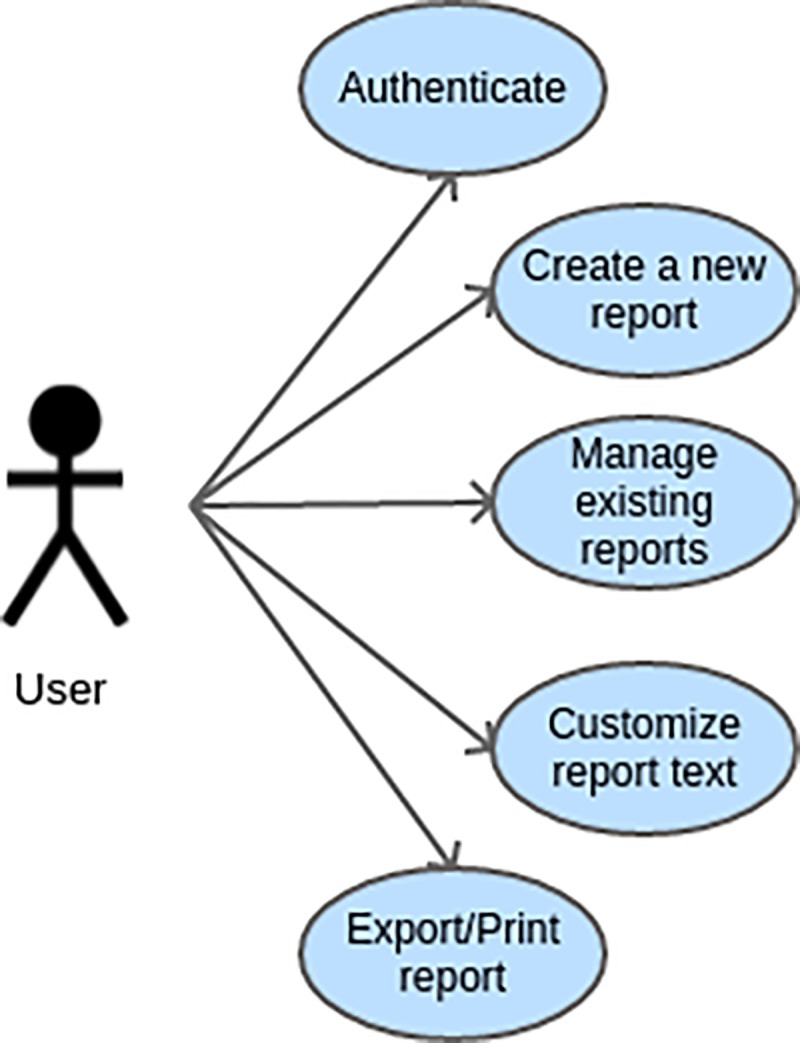
Use case diagram.

Thus, the Use Case Diagram below describes the functionality of the AIPE Diagnostic Report system that is available to the application user (Fig 1).

### 2.2 System architecture

The software was developed following the client-server architecture standard, so that the features implemented in the AIPE diagnostic reports application may be available to any user with internet access.

The implementation of this architecture took place on a platform known as LAMP, which consists of joining the components Linux (Operating System), Apache (Web Server), MySQL (Database) and PHP (Programming Language). This platform was employed because it is a consolidated model in the market to enable the development of web applications, facilitating the management of topics such as security, administration and maintenance of the application.

## 3. Development

The entire application was developed through the PHP, Javascript / Jquery, HTML and CSS languages, using the Bootstrap framework to build responsive pages (that is, pages that visually respond to the user screen resolution), Mysql database for storing report entries and open source libraries like FPDF (to generate PDF reports) and Gentetella Alela (Bootstrap administration page template).

All application source code has been moved to the hosting server and is not available in any code repository like Git, for example.

## 4. Testing

During the development of the software, a Beta model was tested by 30 professional experts in aesthetic damage analysis. These professionals were randomly selected from a list of 100 specialists that have expertise on corporal damage. They were trained at the University of São Paulo, School of Dentistry–FOUSP, Social Dentistry Department and were instructed to compare the use of paper and tables with the software to analyze aesthetic damage. They evaluated the assessment speed for each method and ease and difficulties of using the software in real cases involving the aesthetic damage. After 60 days 17 professionals returned the surveys. Most of them reported the ease and decrease of the analysis time compared to the paper and tables method. Other suggestions have been incorporated in the system. There is also an e-mail at the first page of the website so users can contact the developers with questions or suggestions regarding the system.

The main suggestions are described below at Table 2.

**Table 2:** Professionals’ surveys and suggestions incorporated in the system.

Suggestions/Professionals	1	2	3	4	5	6	7	8	9	10	11	12	13	14	15	16	17
A-Decrease in evaluation time	X	X	X	X	X	X	X	X	X	X	X	X		X	X	X	X
B-Easy to use	X	X	X		X		X	X	X		X	X		X	X	X	
C-Remove text boxes with distances (intimate relationship) on step 2 description	X		X		X			X									
D-Remove text boxes with distances (social relationship) on step 2 description	X		X		X			X									
E-Insert 50 centimeters distance (intimate relationship) on step 2 description	X		X		X			X									
F-Insert 3 meters distance (social relationship) on step 2 description	X		X		X			X									
G-Insert score for each aesthetic damage category on tables—step 3							X	X		X							
H-Change in date presentation format										X			X				
I- Inclusion of the median region on step 2 description								X		X							
J-Insert others and text box to specify lesion location	X						X										
K-Change color presentation format (use a darker tone)													X				
L-Changing the figures on pages													X				X
M-In conclusion: there are two points	X			X													
N-Removing surfaces (hands and feet) on step 2 description								X									

The software works well in all browsers, however some users described that they had a better experience with Mozilla Firefox ESR.

## 5. Operation

The application is running on Goddady Inc. (www.godaddy.com) hosting server and the current hosting plan has the following features:

### Starter hosting

- 1 CPU
- 512 MB RAM
- 250,000 files (inodes)
- 100 simultaneous connections (entry processes)

The limit of the plan may impact the high availability and performance of the application. However, this is merely a hosting system that may allow the migration to a system with greater capacity to support a greater number of requests if needed.

### Ethics statement

This project was approved by the Committee of Ethics in Research (Internal Review Board–IRB) of the University of São Paulo–School of Dentistry–FOUSP under number 43009915.2.0000.0075 on 06/24/2015.

## Results

### System skills

The system is able to receive text entries in predetermined information fields; to insert the information obtained in the base text so as to fill in the gaps in the main text; and to configure an intuitive model of aesthetic damage measurement according to the AIPE methodology.

Text input is enabled through a dynamic and intuitive form, accessible through a common generation and reporting screen.

The software is available in English with the possibility of access in Portuguese and Spanish at the home screen.

### Access control

**Fig 2:**
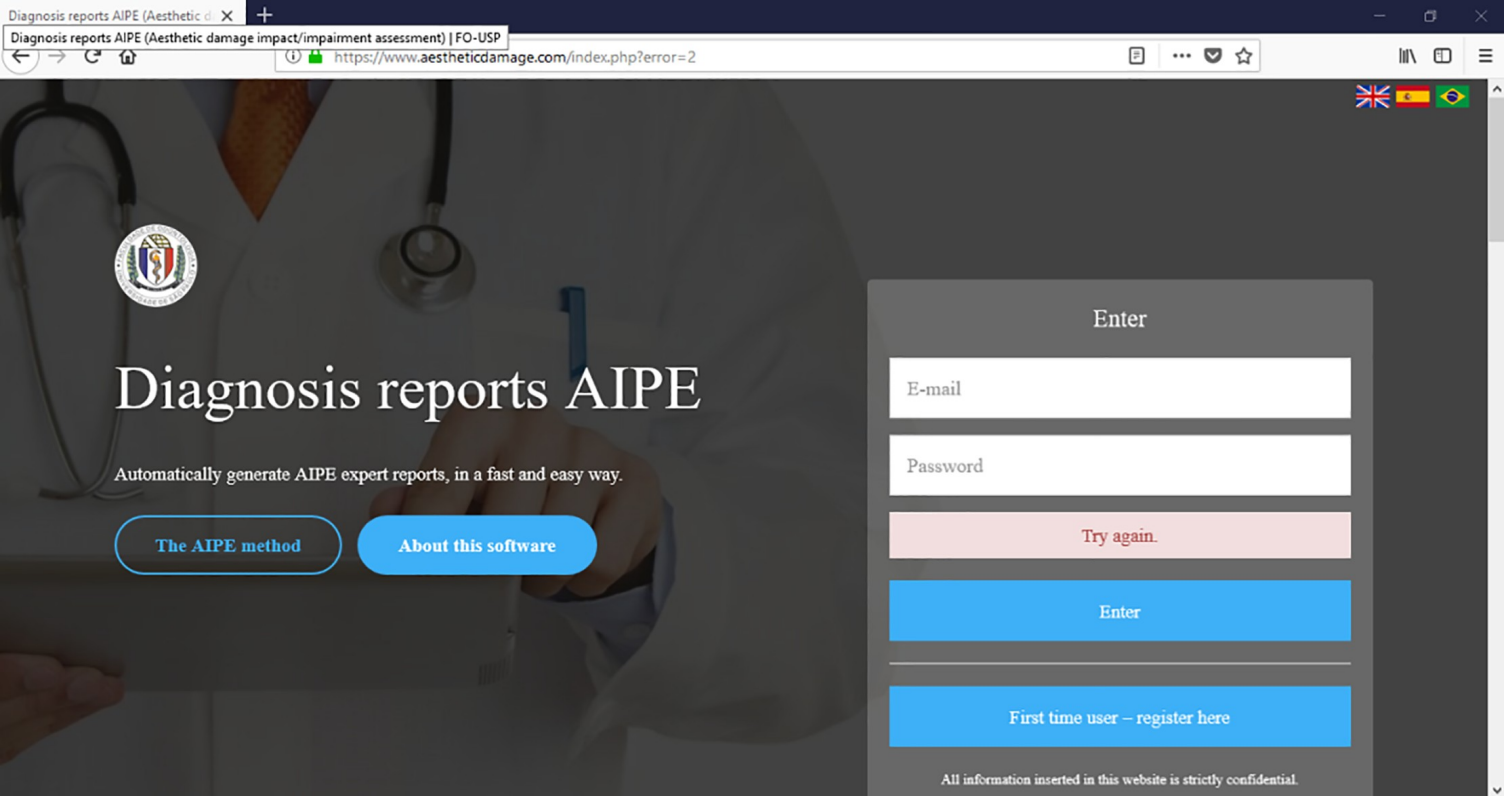
Create new user/ login screen.

User / password system allows only authorized persons to use the program features. This procedure allows superior control over who accesses the software (Fig 2). The implementation of this functionality occurs via a login system. It is essential to emphasize that the entire system is encrypted, i.e. the security of the information entered in the report was a main factor in software development.

The access screen also allows the opening of two explanatory windows regarding both the software operation and the AIPE method [5].

**Fig 3:**
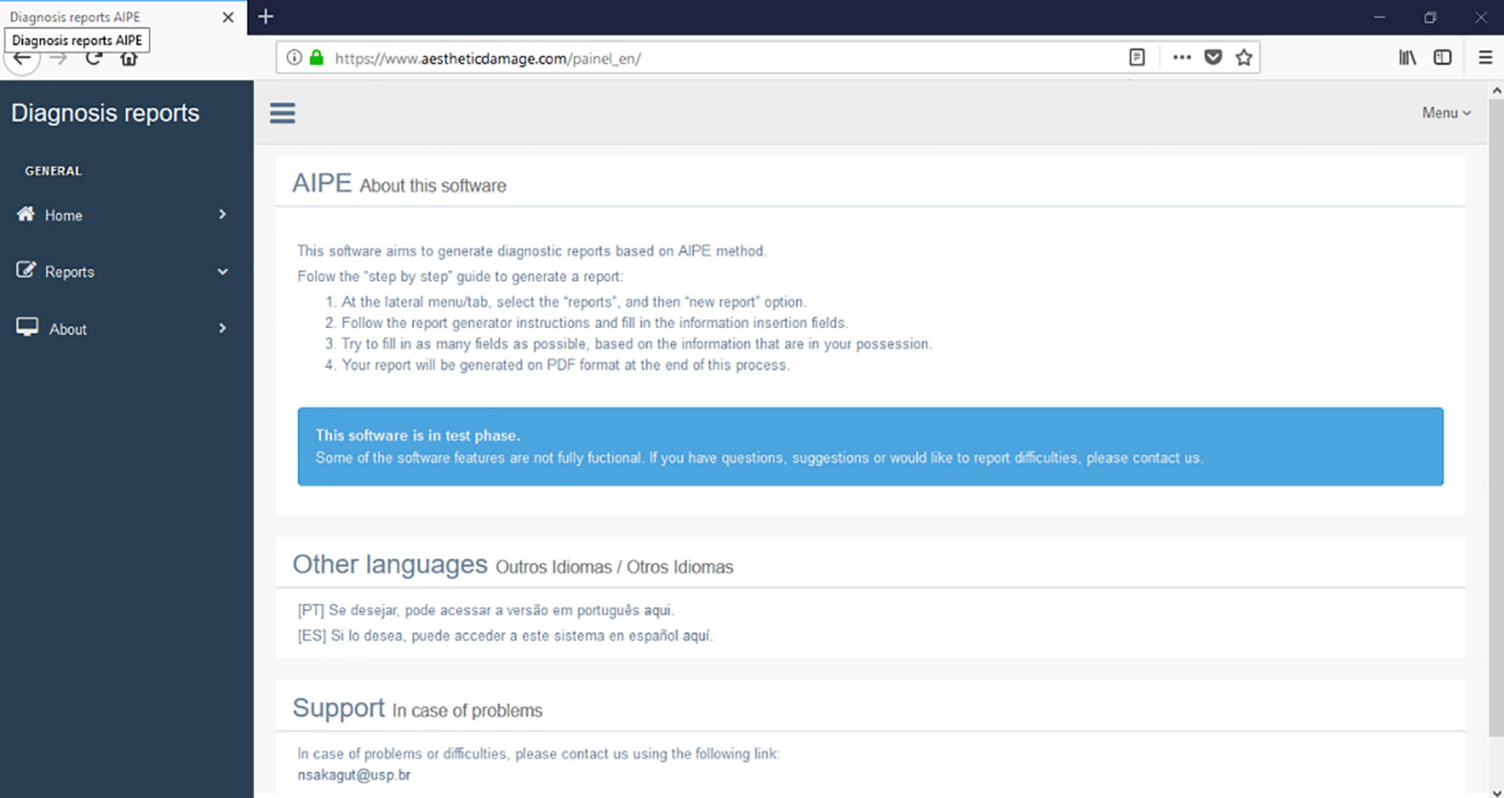
Report generation process initial screen.

At the initial screen of report generation, the user must enter the patient’s or person to be aesthetically evaluated personal data and his/her qualification. Also on this screen, time and place of the exam are recorded. Date is automatically entered by the software (Fig 3).

**Fig 4:**
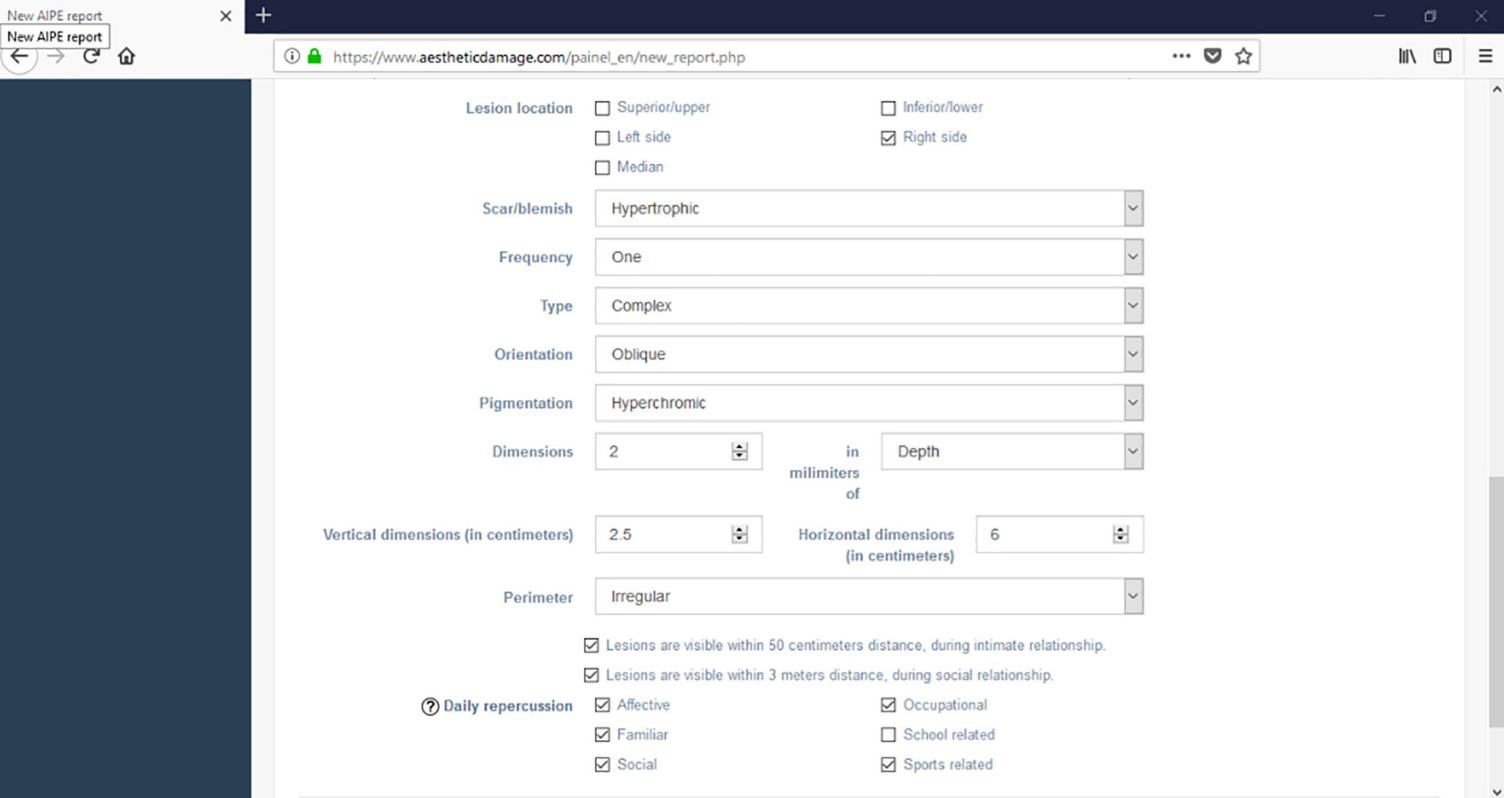
Injury data and social repercussion screen.

At the lesion description screen, one may find relevant information regarding the lesion such as type, location, size, shape, color, elevation and other data. It is also on this screen that personal, professional and social repercussions experienced by the injured person are reported (Fig 4).

**Fig 5:**
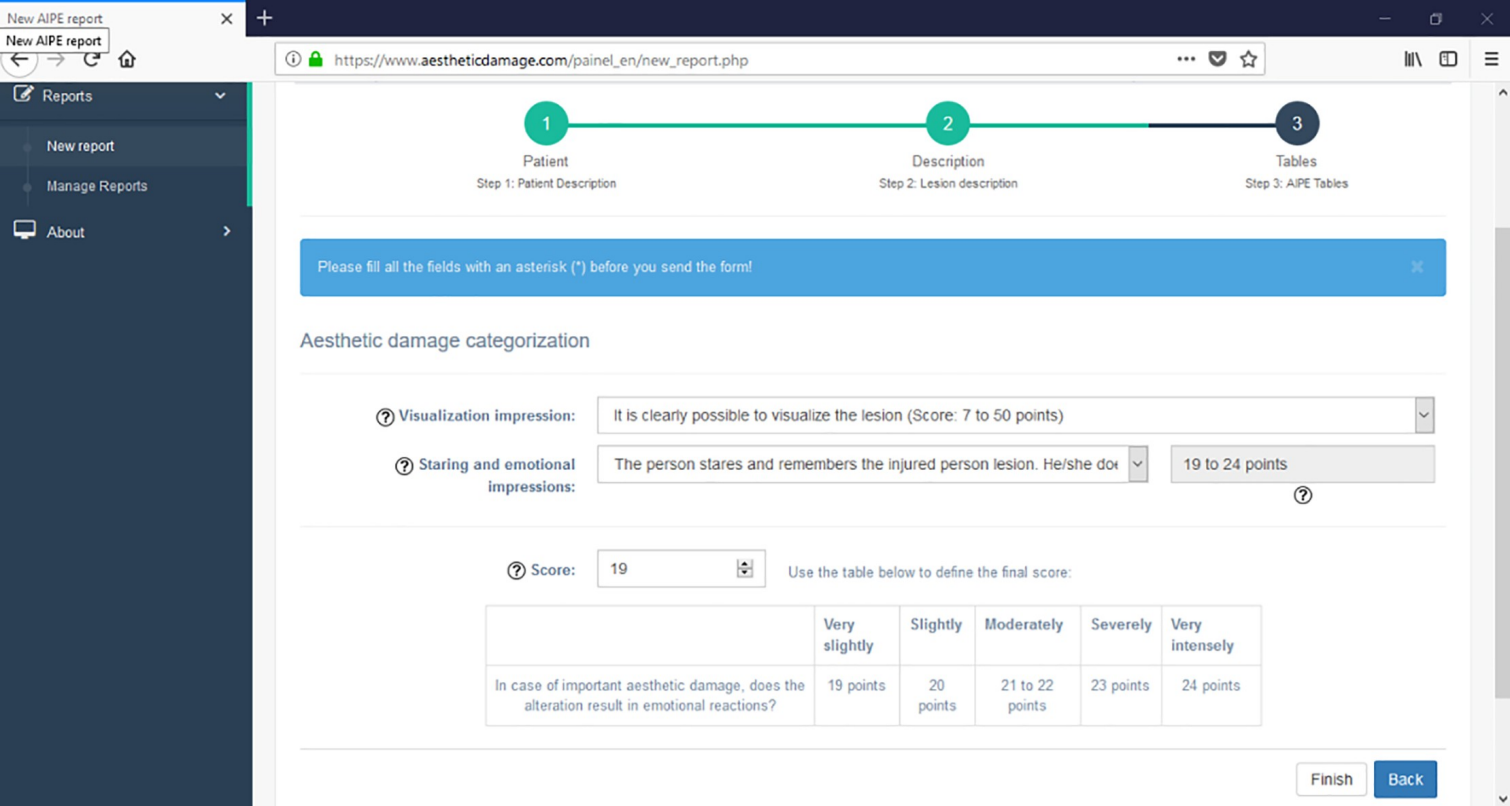
Aesthetic damage qualification screen.

For measuring the aesthetic damage according to the report description, the system uses conditional evaluations that apply the AIPE diagnostic model. In other words, dropdowns that allow the user to select the level of visual evidence of the lesion (s) and then quantify them according to the methodology are employed. There are also aid icons explaining the questions better and what kind of response the users are expected to receive (Fig 5).

**Fig 6:**
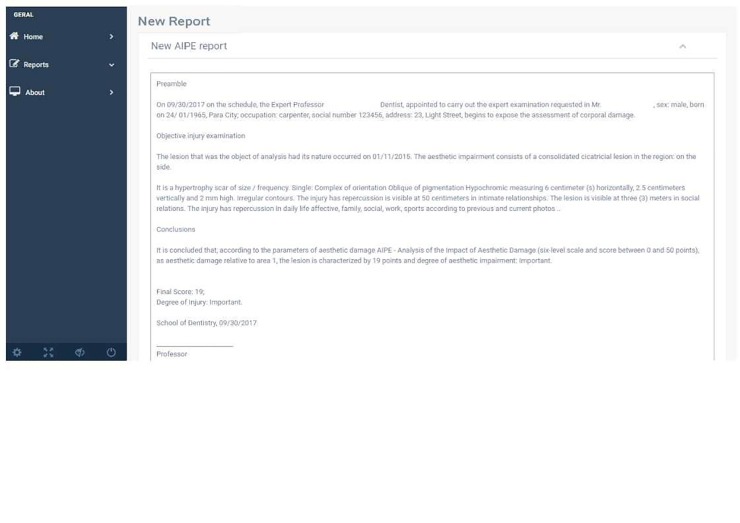
Report completion screen (simulated example).

After both qualification and quantification of the lesion, a base text is generated. This text may be modified after the report is generated, according to particular needs of each evaluation (Fig 6).

### Increments/Additional features

As alternative functionalities to streamline the software scope, some features were added to the system:

#### a) Administrative panel for editing the base text and entries

As it was initially proposed, this functionality would be presented in form of an access system area that was restricted to the system administrator. At that location, one could modify which entries and how much data would be displayed at the base text, as well as the contents of this text. The programmers suggested this functionality was implemented, once diagnosis sometimes is dynamic and may differ depending on the injured person.

After all the information is inserted, the software provides a preliminary report of the opinion describing the injury and the categorization of aesthetic damage (there is no damage, very slightly, slightly, moderately, severely, very intensely). At the text modification screen, the report can be saved in its original form or edited by the user. On this page, the user can insert before and after the injury images to better represent the damage. By pressing the "Report" button, a PDF format file is generated.

#### b) Printing the report

This feature allows the physical printing of the report directly from the software.

**Fig 7:**
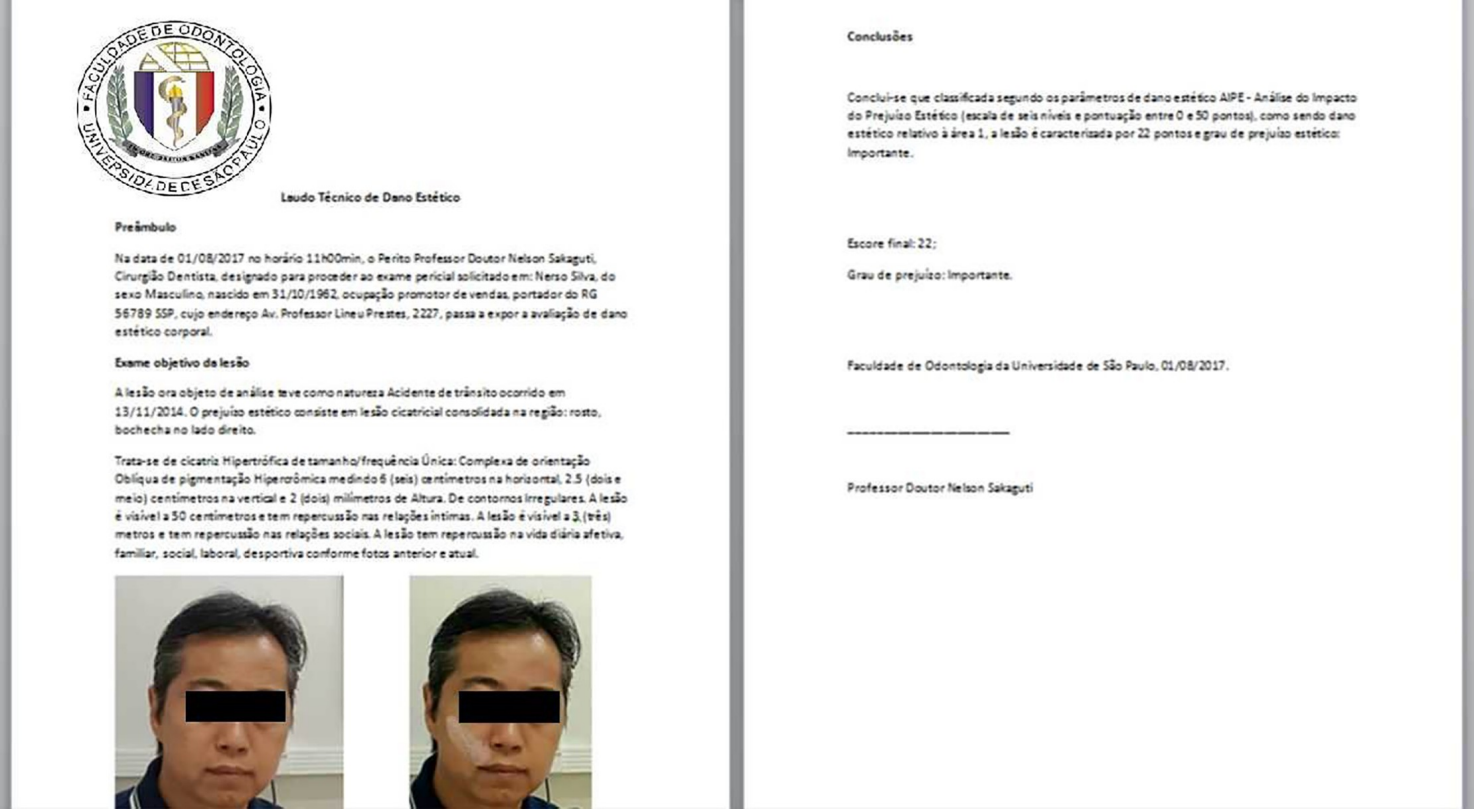
PDF report screen (example of a simulated case report in Portuguese).

At the end of the report generation process, the system creates a PDF report and saves it to the cloud. The user may access the generated report through the "generate reports" section and may insert institutional letterheads by simply making the configuration changes on the page itself (Fig 7).

## Discussion

The quantification of aesthetic damage is complex and one of the main difficulties of health professionals facing a person with bodily injury [3–5]. The use of information technology simplicity is a determining factor that encourages users to accept this mode of knowledge [8]. The challenge was to develop a software operation as simple as possible, whose main functionality is to generate a report of aesthetic damages based on data that were entered by the user.

A research compared the speed of information processing among people using computers with people who use sheets of paper. The study found that using the computer the processing time decreased [9, 14]. The reason for the longer processing time when using the pencil version would be due to the time required to write the response, controlling the correct transfer of the response. Thus using specific software to evaluate an aesthetic damage facilitates the expert’s work, because it is possible to insert the data in an interactive and organized way. Our tests carried out by specialists in assessing aesthetic damage during the software development, also concluded there was a reduction in the evaluation time, since in addition to the data organization, the system does not allow misunderstandings, such as incorrect tables calculations or transfer from one page to another between sheets of paper.

Reducing diagnostic errors has been a goal of medical informatics. In diagnostic tests, the benefits of artificial intelligence software were valued by physicians where they were able to use the additional clinical context for a richer interpretation of the tests. The results showed a generalized agreement, increased integration and workflow and thus the usefulness of the tool [15]. Our system facilitates quantitative assessment as it analyzes all information in an orderly and increasing manner according to the degree and severity of the aesthetic damage. Information about the injury and its social repercussion are inserted in a form and transformed into a PDF file called "base text".

Because the increased access to high-speed internet, PCs, tablets and smartphones, many professionals have started to use mobile applications (apps) to manage theirs works and various health needs. These devices and mobile apps are now increasingly used and integrated with telemedicine and telehealth reducing working time via Internet [16]. The insertion of this software on the internet, in addition to reducing the amount of time by joining data outputs, also allows end users (specialists) to work anytime, anywhere.

Overall, the assessment of attractiveness is a complex process based on individual experiences and subjective perceptions [17]. In cosmetic surgery and dental aesthetic this evaluation is important for the well-being and health of the patients despite the difficulties [18–20]. Several methodologies were developed to identify ways to use computer technology to simulate human perception and identify patterns for perceived attractiveness. However, different perceptions and opinions on attractiveness and aesthetic damage will always exist, even if these perceptions are "standardized" by computational methods [7]. The system inserts the data in an organized and growing way, independent of the user experience, and so, the software can make the analysis less subjective. This software does not intend to homogenize the opinions of the evaluators, but rather support aesthetic damage evaluation in a way that evaluator has the opportunity to customize his report and also, health professionals may show the beauty increase or decrease to their patients.

## Conclusion

The present study described the development of open-access auxiliary software for the evaluation of aesthetic damage. This software provided the users with an easy to use system, by filling predetermined areas to build an evaluation report. With no cost, it may be accessed by professional experts and health professionals who work with aesthetic damage from anywhere in the world via web at www.aestheticdamage.com. The remote access may be conducted via computer, tablet or smartphone as long as the device is connected to the internet, at any time and with no commitment. The software is available in three languages: English, Spanish and Portuguese. Some software features can be adjusted and the text customized by the user. It also transforms the report into a PDF file and saves all evaluations from the same user under his/her file. Information is encrypted for added security and confidentiality.

## Supporting information

S1 Code site 1.
(ZIP)
