# Supplementary material for: Development of a computer-aided design software for the quantitative evaluation of aesthetic damage
Source: PLoS One. 2019 Dec 18;14(12):e0226322. doi: 10.1371/journal.pone.0226322 (PMC6919621; doi:10.1371/journal.pone.0226322)
Supplement: S1 Code site — 1. (ZIP) [file pone.0226322.s001.zip › [EESC_jr][Projeto]Relatorio_07_21/painel/icons.html]

Gentellela Alela! | 


Gentellela Alela!

Welcome,

## John Doe

  


### General

- Home 
  - Dashboard
  - Dashboard2
  - Dashboard3
- Forms 
  - General Form
  - Advanced Components
  - Form Validation
  - Form Wizard
  - Form Upload
  - Form Buttons
- UI Elements 
  - General Elements
  - Media Gallery
  - Typography
  - Icons
  - Glyphicons
  - Widgets
  - Invoice
  - Inbox
  - Calendar
- Tables 
  - Tables
  - Table Dynamic
- Data Presentation 
  - Chart JS
  - Chart JS2
  - Moris JS
  - ECharts
  - Other Charts
- Layouts 
  - Fixed Sidebar
  - Fixed Footer

### Live On

- Additional Pages 
  - E-commerce
  - Projects
  - Project Detail
  - Contacts
  - Profile
- Extras 
  - 403 Error
  - 404 Error
  - 500 Error
  - Plain Page
  - Login Page
  - Pricing Tables
- Multilevel Menu 
  - Level One- Level One
      - Level Two
      - Level Two
      - Level Two
    - Level One
- Landing Page Coming Soon

- John Doe
  - Profile
  - 50%
    Settings
  - Help
  - Log Out
- 6
  - John Smith
    3 mins ago

    Film festivals used to be do-or-die moments for movie makers. They were where...
  - John Smith
    3 mins ago

    Film festivals used to be do-or-die moments for movie makers. They were where...
  - John Smith
    3 mins ago

    Film festivals used to be do-or-die moments for movie makers. They were where...
  - John Smith
    3 mins ago

    Film festivals used to be do-or-die moments for movie makers. They were where...
  - **See All Alerts**


### Font Awesome Icons

Go!

## Font Awesome Icons different icon design elements

- - Settings 1
  - Settings 2

## Web Application Icons

fa-adjust

fa-anchor

fa-archive

fa-area-chart

fa-arrows

fa-arrows-h

fa-arrows-v

fa-asterisk

fa-at

fa-automobile (alias)

fa-ban

fa-bank (alias)

fa-bar-chart

fa-bar-chart-o (alias)

fa-barcode

fa-bars

fa-beer

fa-bell

fa-bell-o

fa-bell-slash

fa-bell-slash-o

fa-bicycle

fa-binoculars

fa-birthday-cake

fa-bolt

fa-bomb

fa-book

fa-bookmark

fa-bookmark-o

fa-briefcase

fa-bug

fa-building

fa-building-o

fa-bullhorn

fa-bullseye

fa-bus

fa-cab (alias)

fa-calculator

fa-calendar

fa-calendar-o

fa-camera

fa-camera-retro

fa-car

fa-caret-square-o-down

fa-caret-square-o-left

fa-caret-square-o-right

fa-caret-square-o-up

fa-cc

fa-certificate

fa-check

fa-check-circle

fa-check-circle-o

fa-check-square

fa-check-square-o

fa-child

fa-circle

fa-circle-o

fa-circle-o-notch

fa-circle-thin

fa-clock-o

fa-close (alias)

fa-cloud

fa-cloud-download

fa-cloud-upload

fa-code

fa-code-fork

fa-coffee

fa-cog

fa-cogs

fa-comment

fa-comment-o

fa-comments

fa-comments-o

fa-compass

fa-copyright

fa-credit-card

fa-crop

fa-crosshairs

fa-cube

fa-cubes

fa-cutlery

fa-dashboard (alias)

fa-database

fa-desktop

fa-dot-circle-o

fa-download

fa-edit (alias)

fa-ellipsis-h

fa-ellipsis-v

fa-envelope

fa-envelope-o

fa-envelope-square

fa-eraser

fa-exchange

fa-exclamation

fa-exclamation-circle

fa-exclamation-triangle

fa-external-link

fa-external-link-square

fa-eye

fa-eye-slash

fa-eyedropper

fa-fax

fa-female

fa-fighter-jet

fa-file-archive-o

fa-file-audio-o

fa-file-code-o

fa-file-excel-o

fa-file-image-o

fa-file-movie-o (alias)

fa-file-pdf-o

fa-file-photo-o (alias)

fa-file-picture-o (alias)

fa-file-powerpoint-o

fa-file-sound-o (alias)

fa-file-video-o

fa-file-word-o

fa-file-zip-o (alias)

fa-film

fa-filter

fa-fire

fa-fire-extinguisher

fa-flag

fa-flag-checkered

fa-flag-o

fa-flash (alias)

fa-flask

fa-folder

fa-folder-o

fa-folder-open

fa-folder-open-o

fa-frown-o

fa-futbol-o

fa-gamepad

fa-gavel

fa-gear (alias)

fa-gears (alias)

fa-gift

fa-glass

fa-globe

fa-graduation-cap

fa-group (alias)

fa-hdd-o

fa-headphones

fa-heart

fa-heart-o

fa-history

fa-home

fa-image (alias)

fa-inbox

fa-info

fa-info-circle

fa-institution (alias)

fa-key

fa-keyboard-o

fa-language

fa-laptop

fa-leaf

fa-legal (alias)

fa-lemon-o

fa-level-down

fa-level-up

fa-life-bouy (alias)

fa-life-buoy (alias)

fa-life-ring

fa-life-saver (alias)

fa-lightbulb-o

fa-line-chart

fa-location-arrow

fa-lock

fa-magic

fa-magnet

fa-mail-forward (alias)

fa-mail-reply (alias)

fa-mail-reply-all (alias)

fa-male

fa-map-marker

fa-meh-o

fa-microphone

fa-microphone-slash

fa-minus

fa-minus-circle

fa-minus-square

fa-minus-square-o

fa-mobile

fa-mobile-phone (alias)

fa-money

fa-moon-o

fa-mortar-board (alias)

fa-music

fa-navicon (alias)

fa-newspaper-o

fa-paint-brush

fa-paper-plane

fa-paper-plane-o

fa-paw

fa-pencil

fa-pencil-square

fa-pencil-square-o

fa-phone

fa-phone-square

fa-photo (alias)

fa-picture-o

fa-pie-chart

fa-plane

fa-plug

fa-plus

fa-plus-circle

fa-plus-square

fa-plus-square-o

fa-power-off

fa-print

fa-puzzle-piece

fa-qrcode

fa-question

fa-question-circle

fa-quote-left

fa-quote-right

fa-random

fa-recycle

fa-refresh

fa-remove (alias)

fa-reorder (alias)

fa-reply

fa-reply-all

fa-retweet

fa-road

fa-rocket

fa-rss

fa-rss-square

fa-search

fa-search-minus

fa-search-plus

fa-send (alias)

fa-send-o (alias)

fa-share

fa-share-alt

fa-share-alt-square

fa-share-square

fa-share-square-o

fa-shield

fa-shopping-cart

fa-sign-in

fa-sign-out

fa-signal

fa-sitemap

fa-sliders

fa-smile-o

fa-soccer-ball-o (alias)

fa-sort

fa-sort-alpha-asc

fa-sort-alpha-desc

fa-sort-amount-asc

fa-sort-amount-desc

fa-sort-asc

fa-sort-desc

fa-sort-down (alias)

fa-sort-numeric-asc

fa-sort-numeric-desc

fa-sort-up (alias)

fa-space-shuttle

fa-spinner

fa-spoon

fa-square

fa-square-o

fa-star

fa-star-half

fa-star-half-empty (alias)

fa-star-half-full (alias)

fa-star-half-o

fa-star-o

fa-suitcase

fa-sun-o

fa-support (alias)

fa-tablet

fa-tachometer

fa-tag

fa-tags

fa-tasks

fa-taxi

fa-terminal

fa-thumb-tack

fa-thumbs-down

fa-thumbs-o-down

fa-thumbs-o-up

fa-thumbs-up

fa-ticket

fa-times

fa-times-circle

fa-times-circle-o

fa-tint

fa-toggle-down (alias)

fa-toggle-left (alias)

fa-toggle-off

fa-toggle-on

fa-toggle-right (alias)

fa-toggle-up (alias)

fa-trash

fa-trash-o

fa-tree

fa-trophy

fa-truck

fa-tty

fa-umbrella

fa-university

fa-unlock

fa-unlock-alt

fa-unsorted (alias)

fa-upload

fa-user

fa-users

fa-video-camera

fa-volume-down

fa-volume-off

fa-volume-up

fa-warning (alias)

fa-wheelchair

fa-wifi

fa-wrench

## File Type Icons

fa-file

fa-file-archive-o

fa-file-audio-o

fa-file-code-o

fa-file-excel-o

fa-file-image-o

fa-file-movie-o (alias)

fa-file-o

fa-file-pdf-o

fa-file-photo-o (alias)

fa-file-picture-o (alias)

fa-file-powerpoint-o

fa-file-sound-o (alias)

fa-file-text

fa-file-text-o

fa-file-video-o

fa-file-word-o

fa-file-zip-o (alias)

## Spinner Icons

- These icons work great with the `fa-spin` class. Check out the
  spinning icons example.

fa-circle-o-notch

fa-cog

fa-gear (alias)

fa-refresh

fa-spinner

## Form Control Icons

fa-check-square

fa-check-square-o

fa-circle

fa-circle-o

fa-dot-circle-o

fa-minus-square

fa-minus-square-o

fa-plus-square

fa-plus-square-o

fa-square

fa-square-o

## Payment Icons

fa-cc-amex

fa-cc-discover

fa-cc-mastercard

fa-cc-paypal

fa-cc-stripe

fa-cc-visa

fa-credit-card

fa-google-wallet

fa-paypal

## Chart Icons

fa-area-chart

fa-bar-chart

fa-bar-chart-o (alias)

fa-line-chart

fa-pie-chart

## Currency Icons

fa-bitcoin (alias)

fa-btc

fa-cny (alias)

fa-dollar (alias)

fa-eur

fa-euro (alias)

fa-gbp

fa-ils

fa-inr

fa-jpy

fa-krw

fa-money

fa-rmb (alias)

fa-rouble (alias)

fa-rub

fa-ruble (alias)

fa-rupee (alias)

fa-shekel (alias)

fa-sheqel (alias)

fa-try

fa-turkish-lira (alias)

fa-usd

fa-won (alias)

fa-yen (alias)

## Text Editor Icons

fa-align-center

fa-align-justify

fa-align-left

fa-align-right

fa-bold

fa-chain (alias)

fa-chain-broken

fa-clipboard

fa-columns

fa-copy (alias)

fa-cut (alias)

fa-dedent (alias)

fa-eraser

fa-file

fa-file-o

fa-file-text

fa-file-text-o

fa-files-o

fa-floppy-o

fa-font

fa-header

fa-indent

fa-italic

fa-link

fa-list

fa-list-alt

fa-list-ol

fa-list-ul

fa-outdent

fa-paperclip

fa-paragraph

fa-paste (alias)

fa-repeat

fa-rotate-left (alias)

fa-rotate-right (alias)

fa-save (alias)

fa-scissors

fa-strikethrough

fa-subscript

fa-superscript

fa-table

fa-text-height

fa-text-width

fa-th

fa-th-large

fa-th-list

fa-underline

fa-undo

fa-unlink (alias)

## Directional Icons

fa-angle-double-down

fa-angle-double-left

fa-angle-double-right

fa-angle-double-up

fa-angle-down

fa-angle-left

fa-angle-right

fa-angle-up

fa-arrow-circle-down

fa-arrow-circle-left

fa-arrow-circle-o-down

fa-arrow-circle-o-left

fa-arrow-circle-o-right

fa-arrow-circle-o-up

fa-arrow-circle-right

fa-arrow-circle-up

fa-arrow-down

fa-arrow-left

fa-arrow-right

fa-arrow-up

fa-arrows

fa-arrows-alt

fa-arrows-h

fa-arrows-v

fa-caret-down

fa-caret-left

fa-caret-right

fa-caret-square-o-down

fa-caret-square-o-left

fa-caret-square-o-right

fa-caret-square-o-up

fa-caret-up

fa-chevron-circle-down

fa-chevron-circle-left

fa-chevron-circle-right

fa-chevron-circle-up

fa-chevron-down

fa-chevron-left

fa-chevron-right

fa-chevron-up

fa-hand-o-down

fa-hand-o-left

fa-hand-o-right

fa-hand-o-up

fa-long-arrow-down

fa-long-arrow-left

fa-long-arrow-right

fa-long-arrow-up

fa-toggle-down (alias)

fa-toggle-left (alias)

fa-toggle-right (alias)

fa-toggle-up (alias)

## Video Player Icons

fa-arrows-alt

fa-backward

fa-compress

fa-eject

fa-expand

fa-fast-backward

fa-fast-forward

fa-forward

fa-pause

fa-play

fa-play-circle

fa-play-circle-o

fa-step-backward

fa-step-forward

fa-stop

fa-youtube-play

## Brand Icons

- All brand icons are trademarks of their respective owners.
- The use of these trademarks does not indicate endorsement of the trademark holder by Font Awesome, nor vice versa.

#### Warning!

Apparently, Adblock Plus can remove Font Awesome brand icons with their "Remove Social Media Buttons" setting. We will not use hacks to force them to display. Please
report an issue with Adblock Plus if you believe this to be an error. To work around this, you'll need to modify the social icon class names.

fa-adn

fa-android

fa-angellist

fa-apple

fa-behance

fa-behance-square

fa-bitbucket

fa-bitbucket-square

fa-bitcoin (alias)

fa-btc

fa-cc-amex

fa-cc-discover

fa-cc-mastercard

fa-cc-paypal

fa-cc-stripe

fa-cc-visa

fa-codepen

fa-css3

fa-delicious

fa-deviantart

fa-digg

fa-dribbble

fa-dropbox

fa-drupal

fa-empire

fa-facebook

fa-facebook-square

fa-flickr

fa-foursquare

fa-ge (alias)

fa-git

fa-git-square

fa-github

fa-github-alt

fa-github-square

fa-gittip

fa-google

fa-google-plus

fa-google-plus-square

fa-google-wallet

fa-hacker-news

fa-html5

fa-instagram

fa-ioxhost

fa-joomla

fa-jsfiddle

fa-lastfm

fa-lastfm-square

fa-linkedin

fa-linkedin-square

fa-linux

fa-maxcdn

fa-meanpath

fa-openid

fa-pagelines

fa-paypal

fa-pied-piper

fa-pied-piper-alt

fa-pinterest

fa-pinterest-square

fa-qq

fa-ra (alias)

fa-rebel

fa-reddit

fa-reddit-square

fa-renren

fa-share-alt

fa-share-alt-square

fa-skype

fa-slack

fa-slideshare

fa-soundcloud

fa-spotify

fa-stack-exchange

fa-stack-overflow

fa-steam

fa-steam-square

fa-stumbleupon

fa-stumbleupon-circle

fa-tencent-weibo

fa-trello

fa-tumblr

fa-tumblr-square

fa-twitch

fa-twitter

fa-twitter-square

fa-vimeo-square

fa-vine

fa-vk

fa-wechat (alias)

fa-weibo

fa-weixin

fa-windows

fa-wordpress

fa-xing

fa-xing-square

fa-yahoo

fa-yelp

fa-youtube

fa-youtube-play

fa-youtube-square

## Medical Icons

fa-ambulance

fa-h-square

fa-hospital-o

fa-medkit

fa-plus-square

fa-stethoscope

fa-user-md

fa-wheelchair


Gentelella - Bootstrap Admin Template by Colorlib
